# A Rare Cause of Mastitis: Idiopathic Granulomatous Mastitis

**DOI:** 10.5334/jbr-btr.1017

**Published:** 2017-01-10

**Authors:** Michiel Eyselbergs, Inge Verslegers, Mireille Van Goethem, Xuan Bich Trinh, Vasiliki Siozopoulou, Paul Parizel

**Affiliations:** 1 Antwerp University Hospital, BE

**Keywords:** Breast, Granulomatous, Mastitis, ultrasound, MRI

A 26-year-old female patient was referred by her gynecologist to the radiology department for evaluation of the right breast. The patient did not have a relevant medical (gynecological) history. During the last five months, she suffered from recurrent breast abscesses. She was treated several times with broad-spectrum antibiotics and surgical drainage, but without clinical improvement. Physical examination revealed a very tender, inflamed breast during palpation. Routine blood tests were normal.

Ultrasonography of the right breast demonstrated a large heterogeneous echoic mass (Figure A). Although not shown in the figure, peripheral hypervascularity and a fistula to the skin in the medial retroareolar region were seen. In addition, multiple smaller collections were seen throughout the right breast. The overlying skin was thickened, and several enlarged axillary lymph nodes were present. Subsequent contrast-enhanced magnetic resonance imaging (MRI) confirmed multiple peripheral-enhancing collections (Figure B1) with diffusion restriction (Figure B2) and corresponding low ADC values (Figure B3) in the right breast. Also, global asymmetric enhancement of the right breast tissue and overlying skin compared to the left side was observed (Figure B1).

**Figure A:**
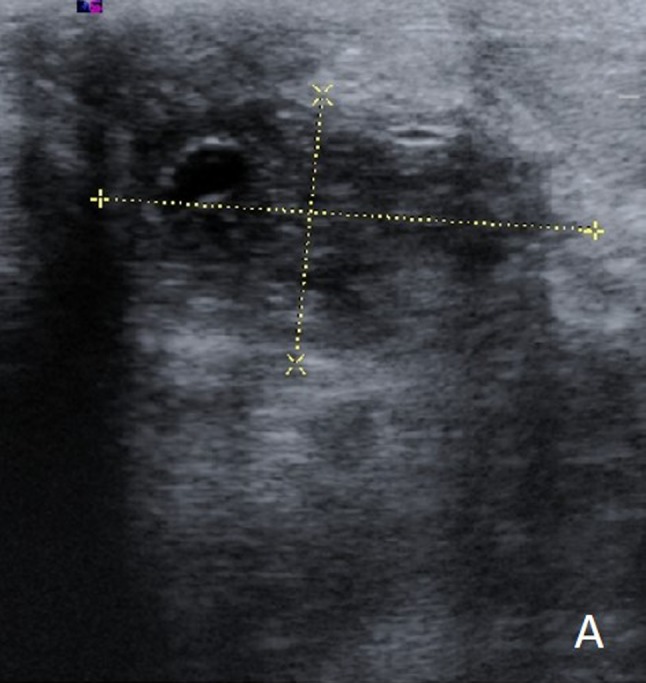


**Figure B:**
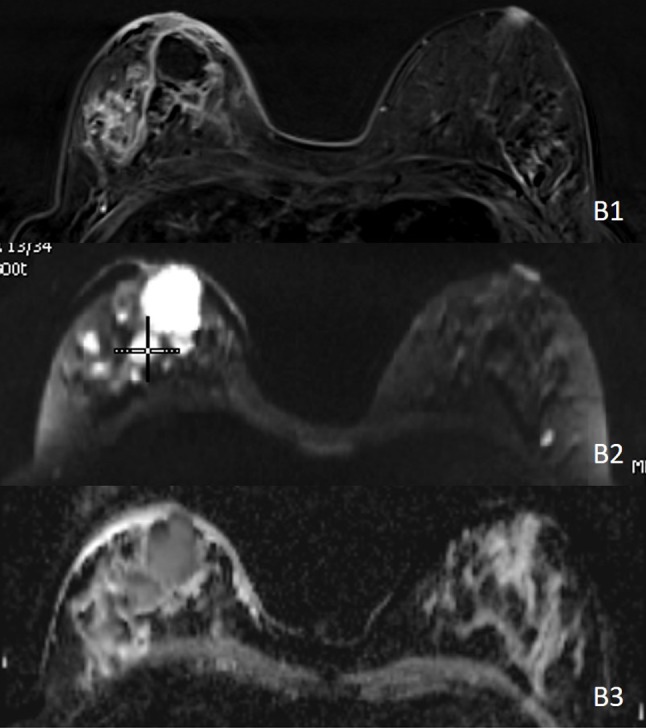


To exclude an underlying carcinoma, a breast biopsy was performed. Malignancy was definitely excluded, but histopathological examination (Figure C) revealed a chronic inflammatory lymphocytic infiltrate (arrow) interspersed with histiocytes (arrowhead) and giant cells (double arrow). No specific aetiological factor could be detected clinically. The patient was treated with corticosteroids, with definite clinical improvement. Based on the clinical history, the imaging features, the histopathology, and good therapeutic response to corticosteroids, the diagnosis of idiopathic granulomatous mastitis (IGM) was made.

**Figure C:**
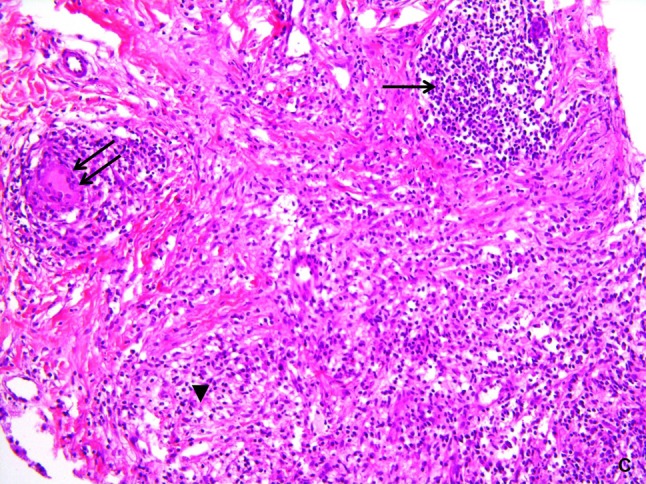


IGM is a very rare chronic inflammatory breast disease. The etiology has not yet been fully elucidated but may be due to an autoimmune process, infection, a chemical reaction associated with oral contraceptive medication, or lactation. Factors such as a hormonal imbalance, autoimmunity, microbiological agents, smoking, and alfa-1 antitrypsin deficiency have also been incriminated recently as causative factors. The term *specific granulomatous mastitis* refers to conditions for which the etiological factor can be identified, and in cases in which this is not, the term *IGM* is used.

IGM typically emerges in woman in the third and fourth decades, although the age range is considerably wider (11–83 years). IGM is usually seen within several years after giving birth and a history of breastfeeding. In contrast, specific GM is more frequently seen in Asian and African countries, with no specific age predilection. The most frequently encountered clinical presentation is a unilateral breast mass nipple retraction and possible sinus formation often associated with inflammation of the overlying skin. Bilateral involvement of the breasts is very rare. This distinct clinico-pathological entity can easily mimic breast carcinoma clinically and radiologically, and the differential diagnosis without histopathological examination is impossible [1].

Imaging features, such as the parenchymal heterogeneity and abscess formation, together with enlarged axillary lymph nodes, support the presence of an inflammatory process. However, these findings are not specific and do not exclude malignancy. Therefore, histopathological confirmation is mandatory to establish the correct diagnosis.
